# Development and Validation of a Consensus-Based Checklist for Regional Anesthesia: The LRA Checklist as a Tool for Safety, Standardization, and Value-Based Care

**DOI:** 10.3390/healthcare14070867

**Published:** 2026-03-27

**Authors:** Antonio Clemente, Domenico Pietro Santonastaso, Mario Bosco, Fabio Costa, Grazia De Angelis, Romualdo Del Buono, Fabio Gori, Giuseppe Lubrano, Valeria Mossetti, Mauro Proietti Pannunzi, Raffaele Russo, Marco Scardino, Giuseppe Sepolvere, Mario Tedesco, Gabriele Melegari, Andrea Tognù, Enrico Barbara, Paolo Grossi, Fabrizio Fattorini

**Affiliations:** 1 Department of Anesthesia and Intensive Care, Santo Spirito in Sassia Hospital, 00193 Rome, Italy; antonio.clemente.anesthesia@gmail.com; 2 Anesthesia and Intensive Care Unit, AUSL Romagna, M. Bufalini Hospital, 47521 Cesena, Italy; d_santonastaso@hotmail.com; 3 Interdepartmental Area of Anesthesia, ASL Roma 1, 00193 Rome, Italy; mario.bosco@aslroma1.it; 4 Unit of Anesthesia and Intensive Care, Fondazione Policlinico Universitario Campus Bio-Medico, 00128 Rome, Italy; f.costa@policlinicocampus.it; 5 Department of Anesthesia and Intensive Care Unit, IRCCS Casa Sollievo Della Sofferenza, 71013 Saint Pio of Pietrelcina, Italy; graziadeangelis22@gmail.com (G.D.A.); rusraf@libero.it (R.R.); 6 Unit of Anesthesia, Intensive Care and Pain Management, ASST (Azienda Socio Sanitaria Territoriale) Gaetano Pini–CTO, 20122 Milan, Italy; romualdodelbuono@gmail.com (R.D.B.);; 7 Anesthesia and Intensive Care Unit, Cardiothoracic, Vascular and Pain Management, Santa Maria della Misericordia Hospital, 06156 Perugia, Italy; fabio.gori@ospedale.perugia.it; 8 Department of Anesthesia and Intensive Care, Ospedale Buonconsiglio Fatebenefratelli, 80123 Naples, Italy; pinolubr@virgilio.it; 9 Pediatric Anesthesia and Intensive Care, Ospedale Infantile Regina Margherita, 10126 Turin, Italy; valeriamossetti@gmail.com; 10 Anesthesia and Intensive Care Service, Casa di Cura “Villa dei Pini”, 62012 Civitanova Marche, Italy; mauro.proietti@kosgroup.com; 11 IRCCS (Istituto di Ricovero e Cura a Carattere Scientifico) Humanitas Research Hospital, 20089 Rozzano, Italy; marco.scardino62@gmail.com; 12 Department of Anesthesia and Cardiac Surgery Intensive Care Unit, Casa di Cura San Michele, 81024 Maddaloni, Italy; giuseppesepolvere@gmail.com; 13 Unit of Anesthesia and Intensive Care, Mater Dei Hospital, 70126 Bari, Italy; mariotedesco@gmail.com; 14 II Service of Anesthesia and Intensive Care, Azienda Ospedaliero-Universitaria Policlinico di Modena, 41121 Modena, Italy; 15 Anesthesia and Intensive Care Unit IC (Istituto Clinico), Humanitas Mater Domini Castellanza, 21053 Varese, Italy; drbarbarbaraenrico@gmail.com (E.B.); pagrossi.dott@gmail.com (P.G.); 16 San Sebastiano Hospital, 00044 Frascati, Italy

**Keywords:** checklist, neurological damage, regional anesthesia, patients’ safety

## Abstract

**Background:** Regional anesthesia is a fundamental aspect of contemporary perioperative care. However, variability in practice, incomplete documentation, and inconsistent safety protocols continue to pose preventable risks. Although there are international checklist models for regional anesthesia and perioperative safety such as those developed by ASRA, ESAIC, and the WHO, Italy does not have a nationally endorsed checklist that is consensus-based and specifically tailored to local terminology, workflows, and legal requirements. **Methods**: To address this gap, we developed an evidence-based Locoregional Anesthesia Checklist (LRA Checklist) using established frameworks for healthcare checklist design. The development process included a needs assessment through a national survey of ESRA Italy members, a review of existing models, item drafting, expert consensus, and endorsement by the Board. We assessed content validity through a modified Delphi process involving 15 experts from the ESRA Italian Chapter Board. Additionally, we created a theoretical impact model to estimate the potential organizational and economic effects of implementing the checklist, using baseline institutional parameters. **Results**: Consensus was achieved for all checklist domains after two Delphi rounds, with minor edits to improve clarity, usability, and clinical relevance. The theoretical model indicates that adopting checklists may help reduce preventable complications, improve workflow, enhance documentation and traceability, and provide overall benefits to institutions in various scenarios. **Conclusions**: In conclusion, the LRA Checklist is a structured, consensus-based tool tailored for the Italian context, aimed at promoting safer and more standardized practices in regional anesthesia. To our knowledge, no prior Italian national consensus or checklist specifically dedicated to regional anesthesia has been formally published. Prospective multicenter studies are necessary to confirm its effectiveness in real-world settings and to quantify both clinical and economic outcomes.

## 1. Introduction

Regional anesthesia (RA) has become an essential component of modern perioperative care, offering effective pain relief, decreasing opioid use, and facilitating early recovery [1,2]. The increasing use of ultrasound guidance, continuous techniques, and multimodal perioperative pathways has significantly improved patient outcomes, but it has also added complexity and medico-legal considerations to regional anesthesia procedures [3,4].

In this evolving landscape, incorporating principles of precision medicine and clinical risk management tools has become fundamental to contemporary anesthetic practice. Over the last decade, the introduction of structured checklists has emerged as an important innovation in surgery, reflecting a broader trend towards safety standardization within perioperative medicine [5]. Checklists serve several purposes: they help prevent avoidable neurological injuries and other complications by ensuring adherence to safety protocols, and they enhance communication among anesthesia and surgical teams [6,7,8].

Their medico-legal significance lies not only in error prevention but also in documenting the safety culture and clinical reasoning that support each procedure. Among the potential adverse events associated with regional anesthesia, neurological injury is considered the most serious and feared complication. Although these injuries are rare, with an estimated incidence of 0.02% to 0.4% depending on the type of block and the patient population, they can lead to prolonged disabilities and legal consequences [9]. The pathophysiology of neurological injuries is often multifactorial, involving mechanical trauma, ischemic injury, or neurotoxicity from local anesthetics. These complications may be exacerbated by inadequate monitoring or insufficient procedural vigilance. Appropriate monitoring, including standard hemodynamic monitoring, ultrasound guidance during block performance, and structured post-procedural observation, can support early detection of local anesthetic systemic toxicity (LAST), vascular puncture, or neurological complications, thereby reducing their severity and clinical impact. Structured checklists can play a vital role in this regard by ensuring systematic pre-procedural verification, appropriate technique selection, continuous patient monitoring, and thorough post-block evaluation [10]. In the Italian healthcare context, variability in documentation practices, organizational models, and medico-legal expectations may further increase the need for structured safety tools. A standardized checklist can improve safety by ensuring consistent adherence to key procedural steps, promoting team communication, strengthening traceability, and supporting risk management. These tools help standardize practices, improve communication among team members, and reduce the likelihood of avoidable neurological damage. Numerous studies have shown that structured checklists can reduce adverse events, such as neurological damage and perioperative morbidity [7]. Likewise, anesthesia-specific checklists have been proven to reduce human error and improve team communication and compliance with safety standards [11]. In this context, the LRA checklist was developed and endorsed by the Board of the Italian chapter of the European Society of Regional Anaesthesia and Pain Therapy. This checklist is intended to guide clinicians through all stages of regional anesthesia, from patient identification and obtaining consent to post-procedural assessment. It incorporates principles of precision medicine, interdisciplinary communication, and clinical risk mitigation. This article presents initial data on the implementation of the LRA checklist at a tertiary university hospital. Additionally, it provides a brief review of existing literature concerning the preventive and legal benefits of utilizing checklist-based safety systems in regional anesthesia.

## 2. Materials and Methods

### 2.1. Phase 1—Preliminary Survey

An initial exploratory survey was distributed to all members of the ESRA Italian Chapter in October 2023 during the ESRA Italian Chapter Meeting in Palermo. The purpose of the questionnaire was to evaluate the perceived need for, and potential usefulness of, a standardized checklist for regional anesthesia procedures within the Italian clinical context. Respondents were asked whether they believed that a checklist was important for enhancing safety, documentation, and training during the execution of both peripheral and central nerve blocks.

### 2.2. Phase 2—Working Group Formation and Review of International Models

To ensure content validity and expert consensus on the checklist items, a modified Delphi process was conducted. Fifteen members of the ESRA Italian Chapter Board (2024) with recognized expertise in regional anesthesia independently evaluated each checklist item using a 5-point Likert scale (from 1 = “not relevant” to 5 = “highly relevant”). Agreement of ≥75% on scores ≥4 was predefined as the threshold for consensus. After the first round, aggregated anonymized feedback was shared with the panel, and a second evaluation round was performed to refine items and enhance clarity and clinical applicability. The process resulted in minor modifications and confirmed the clinical relevance, clarity, and feasibility of all checklist domains. This validation approach ensured the checklist’s methodological rigor and supported its adoption for pilot testing in Italian university hospitals. The LRA checklist was developed in accordance with internationally recognized frameworks for checklist creation in healthcare [12,13,14,15]. The process was structured into six key steps as reported in Table 1.

This structured approach ensured that the LRA Checklist was evidence-based, contextually adapted, and consistent with principles of clinical risk management and medico-legal traceability.

### 2.3. Phase 3—Adaptation to the Italian Context

The structure and content of the ASRA checklist were carefully analyzed to ensure alignment with Italian regulatory standards, clinical practices, and medico-legal requirements. Modifications were made to incorporate national terminology, procedural workflows, and institutional safety protocols commonly used in Italian hospitals. The resulting draft checklist was designed to address all procedural phases, from patient identification and informed consent to post-procedural monitoring, while remaining concise and user-friendly.

### 2.4. Phase 4—Validation by ESRA Italian Chapter Board

**Figure 1:**
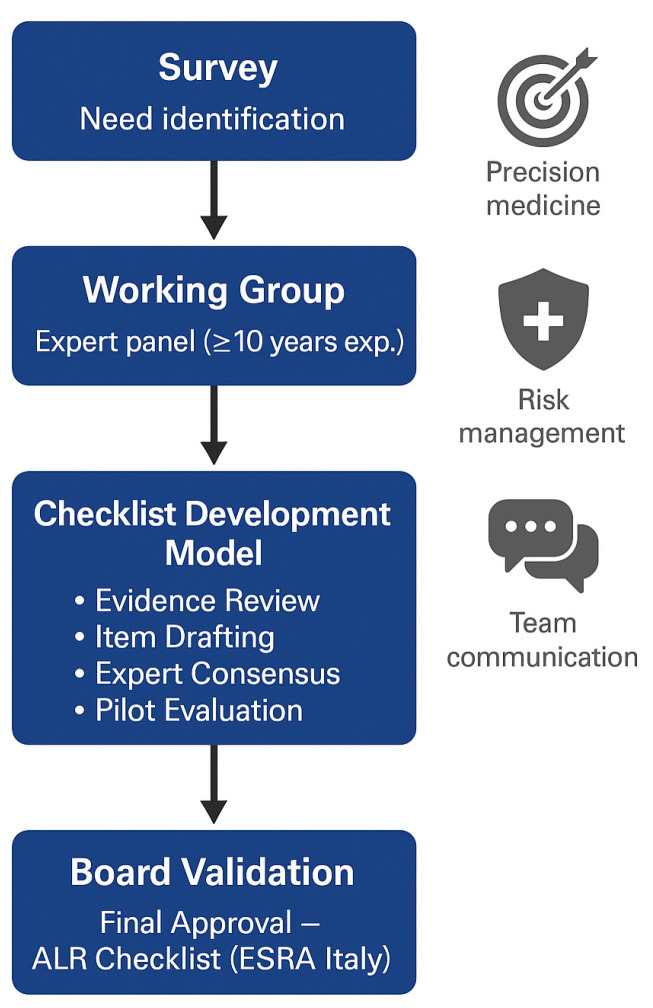
Steps of development of the checklist.

The adapted version of the LRA checklist was then submitted for critical evaluation and approval by the ESRA Italian Chapter Board. Board members reviewed the document for clinical completeness, clarity, and feasibility of implementation. Minor revisions were made based on the Board’s feedback, resulting in a final version approved for dissemination and pilot testing in selected university hospitals as reported in Figure 1.

### 2.5. Preliminary Survey and ESRA Italian Chapter Board Evaluation

Before the Board evaluation, the perceived need for a standardized regional anesthesia checklist was assessed through an exploratory survey distributed to all ESRA Italian Chapter members. The panel members were selected among the ESRA Italian Chapter Board based on predefined criteria, including recognized clinical expertise in regional anesthesia, active practice in high-volume centers, involvement in education and guideline development, and representation of both academic and non-academic institutions across different geographic regions of Italy. All experts had more than 10 years of clinical experience in regional anesthesia. The composition of the panel aimed to ensure diversity of institutional settings while maintaining high-level technical competence, thereby minimizing potential selection bias and enhancing content validity. After the first round of reviews, we analyzed the aggregated anonymized feedback and made minor adjustments to enhance clarity and usability. These changes included refining the wording to eliminate ambiguity, clearly specifying the selected documentation fields, and ensuring consistency in terminology across all sections of the checklist. The questionnaire aimed to evaluate clinicians’ opinions on the utility, feasibility, and perceived impact of implementing a structured checklist in daily practice.

## 3. Results

A total of 144 respondents participated, representing 102 different institutions.

The majority expressed a high level of agreement regarding the importance of introducing a structured tool to enhance safety, documentation accuracy, and teaching consistency. The evaluation involved 15 board members from 15 different Italian hospitals and universities.

Specifically, 100% of respondents considered the checklist essential for improving patient safety and reducing avoidable errors, while 75% viewed it as a valuable support for training residents in regional anesthesia techniques

Several respondents also emphasized its potential medico-legal value, particularly for documenting adherence to institutional safety protocols (see also Supplementary Materials).

**Figure 2:**
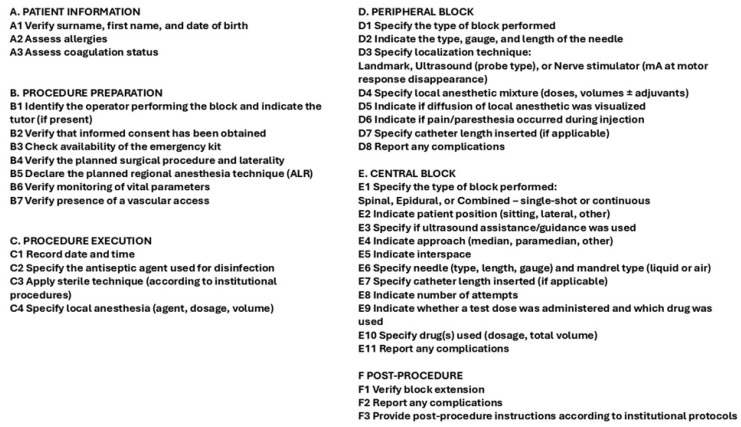
Locoregional Procedure Checklist.

Following this positive feedback, the preliminary version of the “Anestesia Locoregionale (LRA)” Checklist (Figure 2) was developed and submitted to the ESRA Italy Board for critical review. 

### 3.1. Theoretical Impact Model

To complement the qualitative assessment, a simplified theoretical model was developed to estimate the potential organizational and economic impact of implementing the LRA checklist in clinical practice The model was conceived as a pragmatic and reproducible framework to explore how checklist adoption might translate into time optimization, complication reduction, and cost containment, without requiring complex statistical inputs. The approach was designed to remain simple and adaptable, allowing institutions to reproduce the analysis using a limited number of variables, including annual procedure volume (N), staff cost per minute, estimated time savings, baseline complication rate, expected relative risk reduction, and average complication management cost.

The resulting economic effect was computed through the following expression:

Net Benefit = N × [(Cerror × ΔPerror) + (Ctime × ΔT) + Cdoc − Crun] − Cimpl

where

- N = number of regional anesthesia procedures performed per year;

- $C_{\mathrm{error}}$ = mean cost per complication;

- $\Delta P_{\mathrm{error}}$ = absolute reduction in complication probability;

- $C_{\mathrm{time}}$ = staff cost per minute;

- ΔT = net procedural time saved (minutes);

- $C_{\mathrm{doc}}$ = average medico-legal benefit from improved documentation;

- $C_{\mathrm{run}}$ = marginal cost per checklist use;

- $C_{\mathrm{impl}}$ = one-off cost for checklist implementation and training.

A simple one-way sensitivity analysis was performed to explore the robustness of the theoretical model. Key assumptions were varied within plausible ranges, including the relative reduction in complication-related costs (20–40%), net time saved per procedure (1–3 min), and annual procedural volume in scaling scenarios. Under conservative assumptions, the model remained generally neutral-to-favorable, with estimated annual net benefits ranging approximately from €5000 to €18,000 (for 1000 procedures/year). These calculations should be interpreted as illustrative rather than predictive, as real-world effects will depend on implementation quality, adherence, and local institutional context (Table 2 case scenario).

For this theoretical model, “preventable complications” are defined as adverse events that could potentially be mitigated through adherence to standardized safety procedures outlined in the checklist. These complications include, but are not limited to: local anesthetic systemic toxicity (LAST), procedures performed on the incorrect site or for the wrong indication, dosage miscalculations, failure to recognize early neurological symptoms, and documentation errors that affect medico-legal accountability.

“Workflow optimization” refers to potential improvements in procedural efficiency that stem from a structured sequence of safety checks, standardized communication, and the elimination of redundant documentation steps. These concepts serve as parameters for theoretical modeling and were not directly measured as clinical endpoints in this study.

### 3.2. A Case Scenario

A baseline scenario was simulated using conservative and realistic assumptions for a tertiary university hospital:

The model suggests that checklist implementation may generate incremental efficiency gains and risk reduction benefits. However, these estimates are illustrative rather than predictive. The primary value of the model lies in providing a structured framework for institutional evaluation rather than precise economic forecasting. These findings indicate that even modest improvements in procedural safety and the standardization of workflows can provide significant economic and organizational benefits for healthcare institutions.

### 3.3. Interpretation

The model demonstrates that the implementation of the LRA checklist can be economically advantageous, primarily through improved efficiency and prevention of adverse events.

Its simplicity makes it a practical decision-support tool for hospitals seeking to quantify the benefits of structured safety interventions in regional anesthesia.

Although theoretical, these results align with published evidence showing that checklist-based safety systems improve perioperative outcomes and reduce overall healthcare costs.

**Figure 3:**
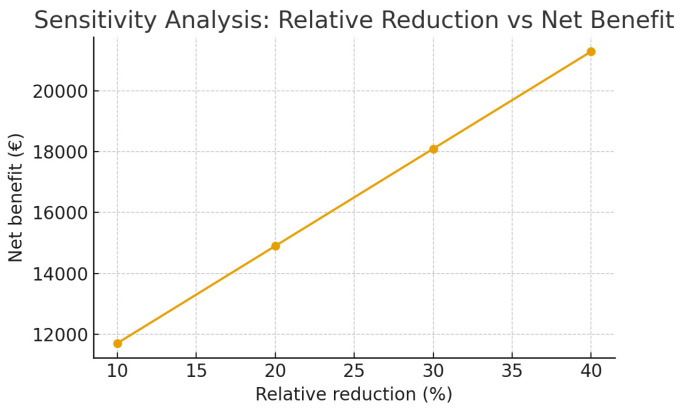
Theoretical Impact Model.

Prospective multicenter validation will be necessary to confirm the model’s predictive reliability in real-world settings (Figure 3).

## 4. Discussion

Checklists are widely acknowledged as crucial safety tools in high-risk industries. They promote standardization, reduce human error, and improve team communication. In aviation, for example, checklists are a fundamental part of daily operations. They ensure consistent performance and help prevent rare but potentially catastrophic incidents. This emphasis on checklists is not a reflection of professionals’ lack of competence; rather, even highly skilled experts benefit from structured cognitive aids in complex environments [16,17]. In recent years, the healthcare sector has increasingly adopted safety strategies similar to those used in aviation. The WHO Surgical Safety Checklist has shown significant improvements in perioperative outcomes and team coordination across various clinical settings. Regional anaesthesia shares many characteristics with aviation; it is a high-skill discipline that requires precise execution under time pressure and involves low-incidence but high-impact risks. Therefore, a structured checklist should be regarded as a practical safety tool rather than merely an administrative formality. It reinforces good clinical practices and helps clinicians provide reliable and consistent care [5,18]. Italian data further support the safety profile of regional anesthesia. The RICALOR prospective registry, involving 17 Italian hospitals and over 117,000 procedures, reported that regional anesthesia represented 54.3% of anesthetic techniques. The overall complication rate was 4.6 per 10,000 procedures, while major complications were extremely rare (0.07/1000). These findings confirm that regional anesthesia is widely practiced in Italy with a high safety profile, but also underline the importance of continuous monitoring and structured reporting systems to maintain and improve patient safety [19].

Among the potential complications of regional anaesthesia, neurological injury is one of the most serious and concerning adverse events. Although these injuries are uncommon, with an estimated incidence of approximately 0.02% to 0.4% depending on the type of block and the patient population, they can lead to long-lasting functional consequences and have medico-legal implications for both clinicians and institutions [16,17]. The pathophysiology of neurological injury is multifactorial, involving aspects such as mechanical trauma, ischemic or inflammatory mechanisms, local anaesthetic neurotoxicity, or a combination of these factors. The risk of these injuries can be exacerbated by inadequate monitoring or suboptimal techniques. A crucial safety aspect that warrants attention is the prevention of Local Anaesthetic Systemic Toxicity (LAST), a rare but potentially life-threatening complication of regional anesthesia. A structured checklist may also contribute to reducing the risk and LAST and neurotoxicity. While a checklist cannot eliminate pharmacological risk, it can promote adherence to key safety behaviors such as dose verification, awareness of cumulative dosing, incremental injection with aspiration, appropriate monitoring, and readiness for emergency management. In addition, checklist-driven workflows may facilitate early recognition of prodromal LAST symptoms and prompt treatment, which are crucial determinants of patient outcomes [16,20]. Despite advancements in ultrasound guidance and dose-limiting strategies, LAST continues to be reported in various clinical settings, often resulting from inaccurate dosing, inadvertent intravascular injection, or delayed recognition of early warning symptoms [21]. To enhance clinician vigilance throughout all procedural phases, it is beneficial to incorporate structured checklist elements specifically designed to prevent LAST. Key preventive measures should include systematic dose calculations based on patient factors, aspiration before injection, incremental dosing with frequent verbal communication about cumulative volume, and real-time monitoring to detect neurological or cardiovascular warning signs [22]. Furthermore, integrating explicit reminders to confirm the immediate availability of lipid emulsion therapy and the presence of a skilled response team reinforces institutional readiness for emergency management. Checklists serve not only as cognitive aids but also as educational tools that promote a culture of safety, consolidating good practices and ensuring team alignment around shared safety standards. Therefore, their role extends beyond mere documentation, becoming an integral component of harm-reduction strategies designed to minimize the incidence and severity of LAST [23].

The theoretical model proposed in this study offers a practical and organized approach to quantifying the potential organizational, safety, and economic benefits of implementing the LRA checklist in clinical practice, as suggested by Mulroy et al. [5]. One of its key strengths is its simplicity and replicability. The model relies on a limited number of easily obtainable institutional variables, including annual procedural volume, staff cost per minute, estimated time savings, baseline complication rate, expected relative risk reduction, and cost of complications. This allows hospitals with different organizational structures to adapt to their local context without requiring complex statistical expertise. Additionally, the model provides a transparent framework that links the use of checklists to measurable outcomes. This transparency facilitates communication with hospital management and supports informed decision-making regarding resource allocation and patient safety investments. Importantly, the checklist underwent a structured content validation through a modified Delphi process involving 15 experts from the ESRA Italian chapter Board, which enhances its methodological rigor and supports the content validity of the tool. However, several limitations should be acknowledged. First, the model is based on theoretical assumptions and preliminary estimates derived from literature-based ranges and expert opinions; therefore, real-world data may vary across institutions. The projected reductions in complications and time savings, while consistent with published evidence on perioperative safety tools, may either underestimate or overestimate the true impact depending on baseline compliance with safety standards and staff engagement. Second, quantifying the economic value of medico-legal benefits is challenging due to variability in institutional risk management policies and national legal frameworks. Third, the model primarily focuses on direct and short-term outcomes, while long-term effects, such as cultural change, improved team performance, enhanced training quality, and patient-reported outcomes, are not addressed [16]. Finally, external validation in diverse clinical settings, ideally through multicenter prospective studies, will be essential to assess generalizability, confirm cost-effectiveness, and refine model parameters.

### 4.1. Perspectives: Implementation Strategy

Successful integration of the LRA checklist into routine clinical practice requires a structured implementation strategy that focuses on training, workflow alignment, and sustained staff engagement. Before deployment, it is essential to conduct targeted education sessions for anesthesiologists, residents, and nursing staff to ensure they understand the checklist’s structure, objectives, and medico-legal significance. Introducing the checklist through short simulation-based workshops or real-time demonstrations can enhance usability and encourage adherence. Local champions or “checklist facilitators” can support the initial implementation by providing on-site guidance and feedback, especially during the early adoption phase. To minimize workflow disruption and promote consistent use, the checklist should be clearly integrated into existing perioperative pathways, either in paper form or embedded into electronic medical records. Future work should aim to enhance perioperative safety, monitoring, and documentation in regional anesthesia by adopting standardized tools and reporting frameworks. One priority area is the broader implementation of objective injection pressure monitoring devices such as the BSmart™ Injection Pressure Monitor (B. Braun Medical, Melsungen, Germany) and the SAFIRA^®^—SAFer Injection for Regional Anaesthesia system (Medovate Ltd., Cambridge, UK) [24,25,26]. The BSmart™ device provides inline, colour-coded feedback on injection pressure during peripheral nerve blocks, offering an objective and quantifiable alternative to subjective “syringe feel” and facilitating documentation of injection pressures within predefined ranges.

Similarly, the SAFIRA^®^ system integrates a safety mechanism that automatically limits the maximum injection pressure and allows the clinician to control both aspiration and infusion with a single operator workflow, potentially reducing the risk of injection at pressures associated with neural injury.

Regular audits and feedback cycles, along with periodic reviews of checklist items and outcome data, will help monitor compliance and identify areas for improvement. Finally, involving frontline clinicians in ongoing updates will strengthen ownership, reduce resistance, and foster a sustainable safety culture within regional anesthesia practice. An additional important determinant of real-world effectiveness is checklist adherence. The potential clinical and organizational benefits described in the theoretical model assume appropriate and consistent implementation. Variability in compliance rates across institutions may significantly influence outcomes, as demonstrated in other checklist based safety interventions. Future prospective studies should therefore assess not only complication trends but also checklist utilization rates and quality of completion, in order to better understand the relationship between adherence and safety impact.

### 4.2. Strengths and Limitations

The questionnaire consisted of structured multiple-choice items using Likert-scale response options (ranging from strong disagreement to strong agreement). Questions addressed perceived necessity of a regional anesthesia checklist, expected impact on patient safety, educational value, and potential medico-legal benefits. The survey was exploratory and descriptive in nature and was not designed as a validated psychometric instrument. (We added survey results in Supplementary Materials) This study adds to the expanding field of safety standardization in regional anesthesia by introducing a carefully developed checklist and a new method for evaluating its impact in healthcare institutions. One of the study’s key strengths is its dual focus: the LRA checklist was created and validated through expert consensus, while also being situated within a broader framework that considers workflow efficiency, legal protection, and organizational value. By incorporating a theoretical model, the study provides a fresh perspective for decision-makers, which could enhance administrative acceptance, an aspect that is often overlooked in literature on checklists. Model parameters were based on conservative, literature-informed assumptions and institutional plausibility ranges rather than empirical prospective data. The estimated absolute reduction in complication probability (ΔPerror) was modeled using cautious ranges extrapolated from published evidence on checklist-based safety interventions in perioperative settings. The documentation-related benefit (Cdoc) was conceptualized as a proxy for improved medico-legal traceability and risk mitigation, acknowledging that such benefits are difficult to quantify directly. These parameters were intentionally conservative to avoid overestimation of economic impact. The model is therefore intended as a structured decision-support framework rather than a formal cost-effectiveness analysis. A one-way sensitivity analysis was performed to explore the robustness of the model under conservative and plausible variations in key assumptions. In addition to varying the relative reduction in complication-related costs (20–40%), we varied net time saved per procedure (1–3 min) and examined the effect of different annual procedure volumes in scaling scenarios. All other parameters were held constant. This approach allowed assessment of model stability without introducing excessive analytical complexity.

However, the study does have limitations. It does not include real-world implementation data, so the projected benefits remain theoretical. The model also fails to account for cultural, behavioral, or organizational factors that can significantly influence the adoption of safety tools. Furthermore, the checklist was specifically tailored to the Italian healthcare setting, which may limit its generalizability until it is adapted and tested in other healthcare systems. Future research should focus on multicenter validation, assessing user compliance over time, and evaluating the checklist’s impact on clinical outcomes, team performance, and training effectiveness.

## 5. Conclusions

The LRA checklist is a structured tool developed through consensus to improve the safety, standardization, and documentation of regional anesthesia practices. Its creation followed a rigorous methodology, which included expert validation of the content. Additionally, a theoretical model suggested potential organizational and economic benefits for healthcare institutions. While these findings are preliminary, they support the integration of the LRA checklist into routine clinical practice as part of a larger patient safety strategy. The effectiveness of checklist implementation should be evaluated through prospective audits measuring adherence to checklist items, quality of documentation, and trends in complication recognition. Future steps include multicenter implementation studies and quality-improvement initiatives comparing safety indicators before and after checklist adoption, in order to quantify its real-world clinical impact. Further multicenter prospective studies are warranted to validate its real-world effectiveness and assess its impact on clinical outcomes, team performance, and healthcare costs.

## Figures and Tables

**Table 1:** Steps of identification and development of Locoregional Checklist.

Needs assessment: identification of procedural and communication gaps through the national ESRA Italian Chapter member survey.
Evidence review: critical analysis of existing checklists (ASRA, ESAIC, WHO) and current literature on perioperative safety.
Item drafting and selection: formulation of concise, action-oriented statements covering all procedural stages (patient identification, preparation, execution, post-procedure).
Expert consensus: iterative discussions among senior anesthesiologists (>10 years’ experience) to ensure clarity, relevance, and feasibility.
Pilot evaluation: small-scale testing for usability and completeness within simulated or real clinical settings.
Validation and approval: final review and endorsement by the ESRA Italian chapter Board.

**Table 2:** Case scenario example.

Component	Assumption (Conservative)	Rationale
Annual number of RA procedures	1000	Example medium-size hospital volume
Average cost of a complication	€4000	Includes diagnostic workup, treatment, prolonged stay (illustrative estimate)
Baseline complication rate	0.05–0.1%	Consistent with published RA safety data
Relative reduction in complication-related costs	20–40%	Plausible improvement from safety standardization
Estimated annual savings from complication reduction	€4000–€12,000	Range reflecting sensitivity assumptions
Time saved per procedure	1–3 min	Conservative estimate
Monetary value of time saved	€1–€3 per minute	Approximate staff cost valuation
Estimated annual workflow-related savings	€1000–€3000	Based on time savings
Documentation/traceability benefit	€1000–€3000	Risk-management value (illustrative)
Implementation cost (training, integration)	~€2000/year	Initial and maintenance estimate
**Calculated Annual Net Benefit (illustrative range)**	**€5000–€18,000**	Derived from model

## Data Availability

The raw data supporting the conclusions of this article will be made available by the authors on request for privacy reason.

## Supplementary Materials

The following supporting information can be downloaded at: https://www.mdpi.com/article/10.3390/healthcare14070867/s1.
