# Supplementary material for: Development and Validation of a Consensus-Based Checklist for Regional Anesthesia: The LRA Checklist as a Tool for Safety, Standardization, and Value-Based Care
Source: Healthcare (Basel). 2026 Mar 27;14(7):867. doi: 10.3390/healthcare14070867 (PMC13074158; doi:10.3390/healthcare14070867)
Supplement: Supplementary file 1 [file healthcare-14-00867-s001.zip › healthcare-4144655-supplementary.pptx]

## Slide 1
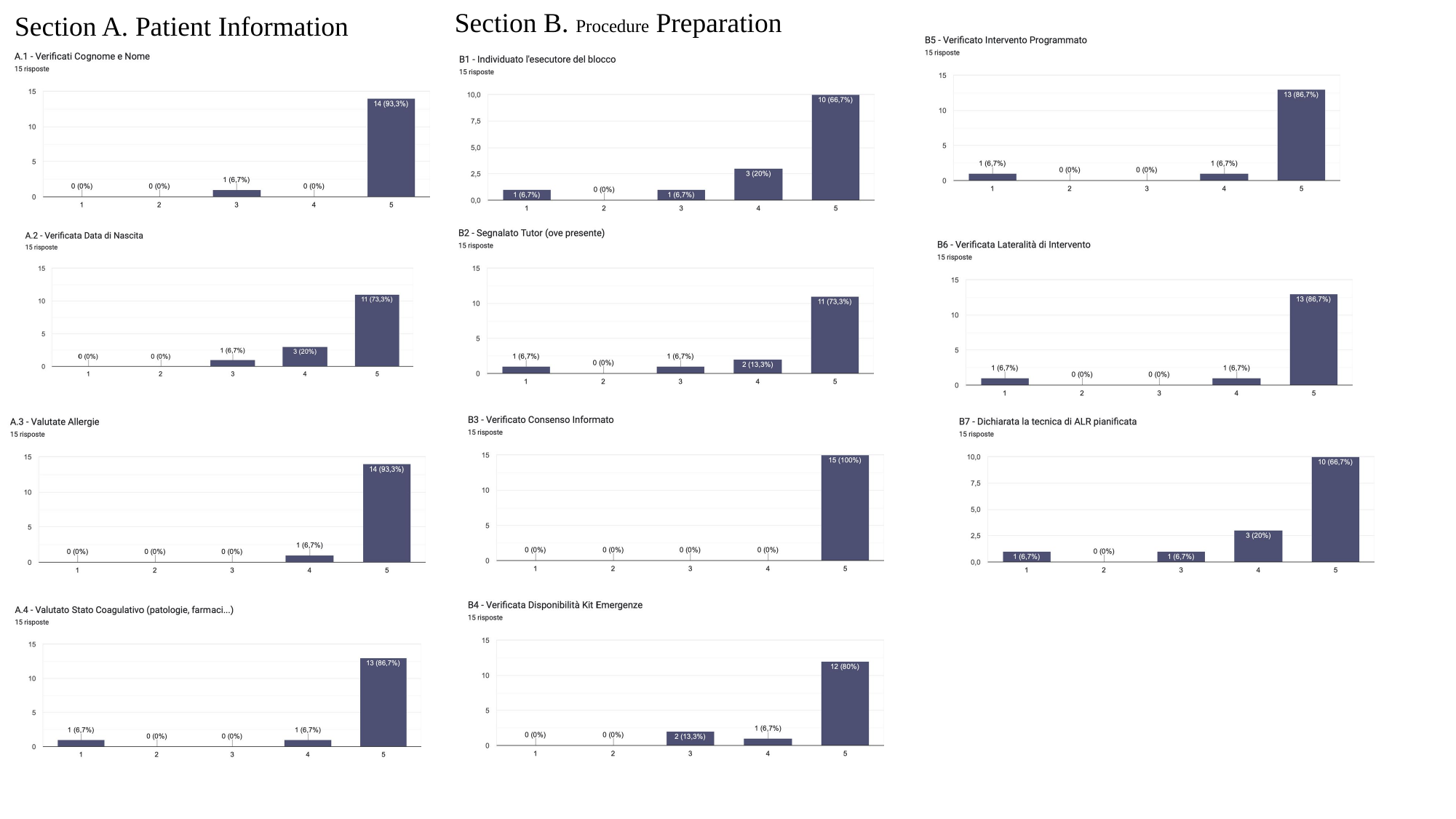

Section B. Procedure Preparation
Section A. Patient Information

## Slide 2
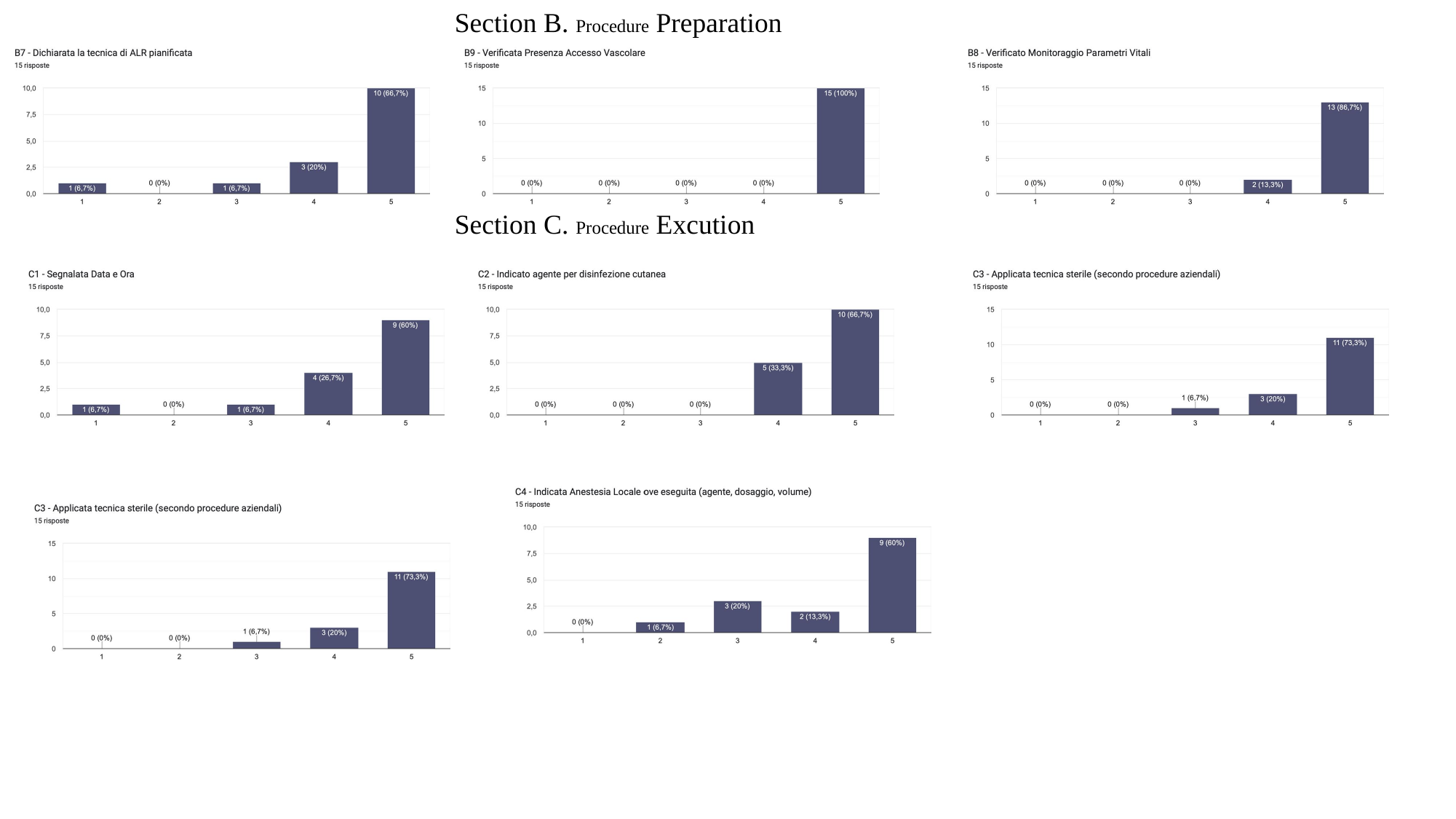

Section B. Procedure Preparation
Section C. Procedure Excution

## Slide 3
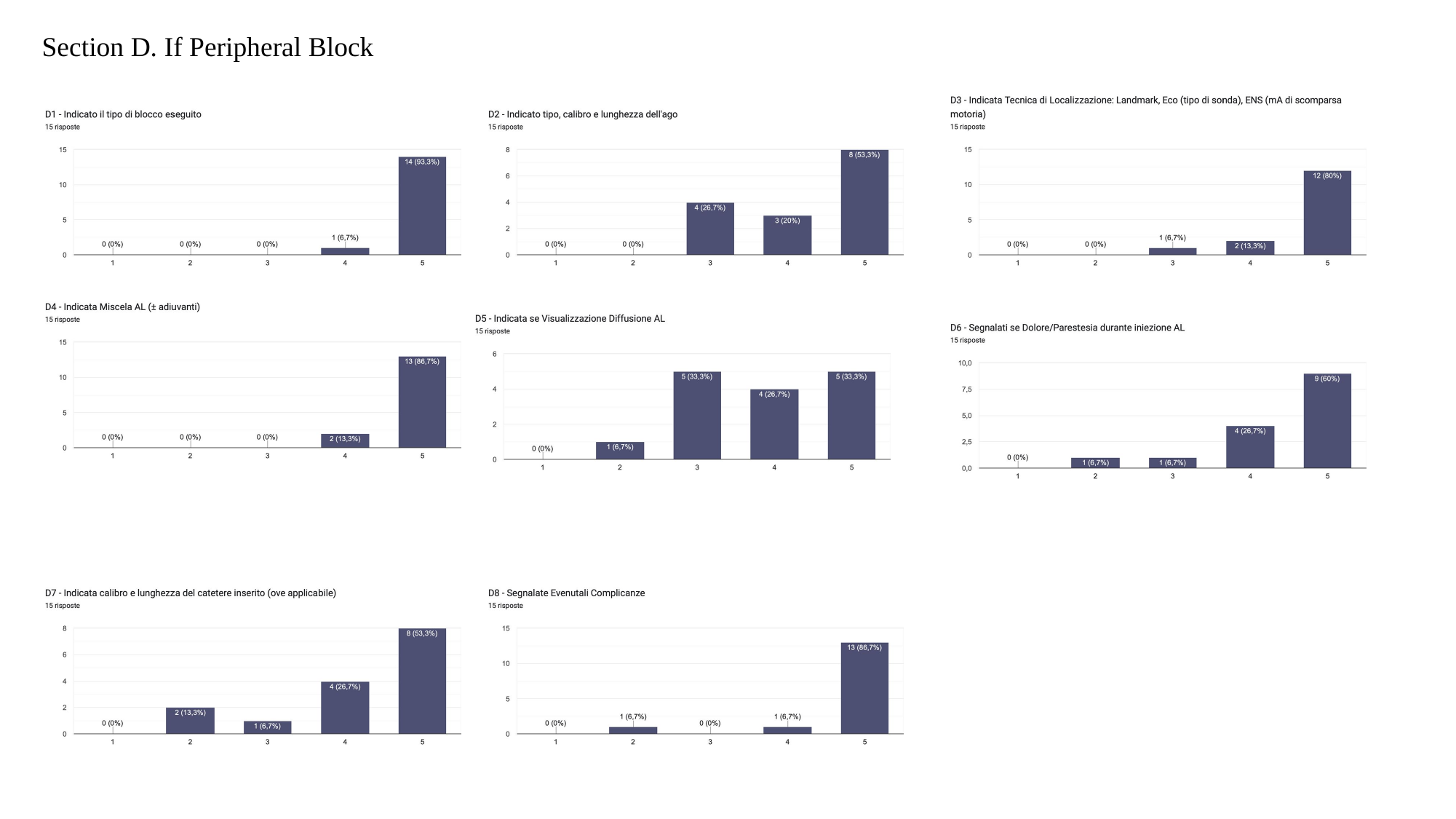

Section D. If Peripheral Block

## Slide 4
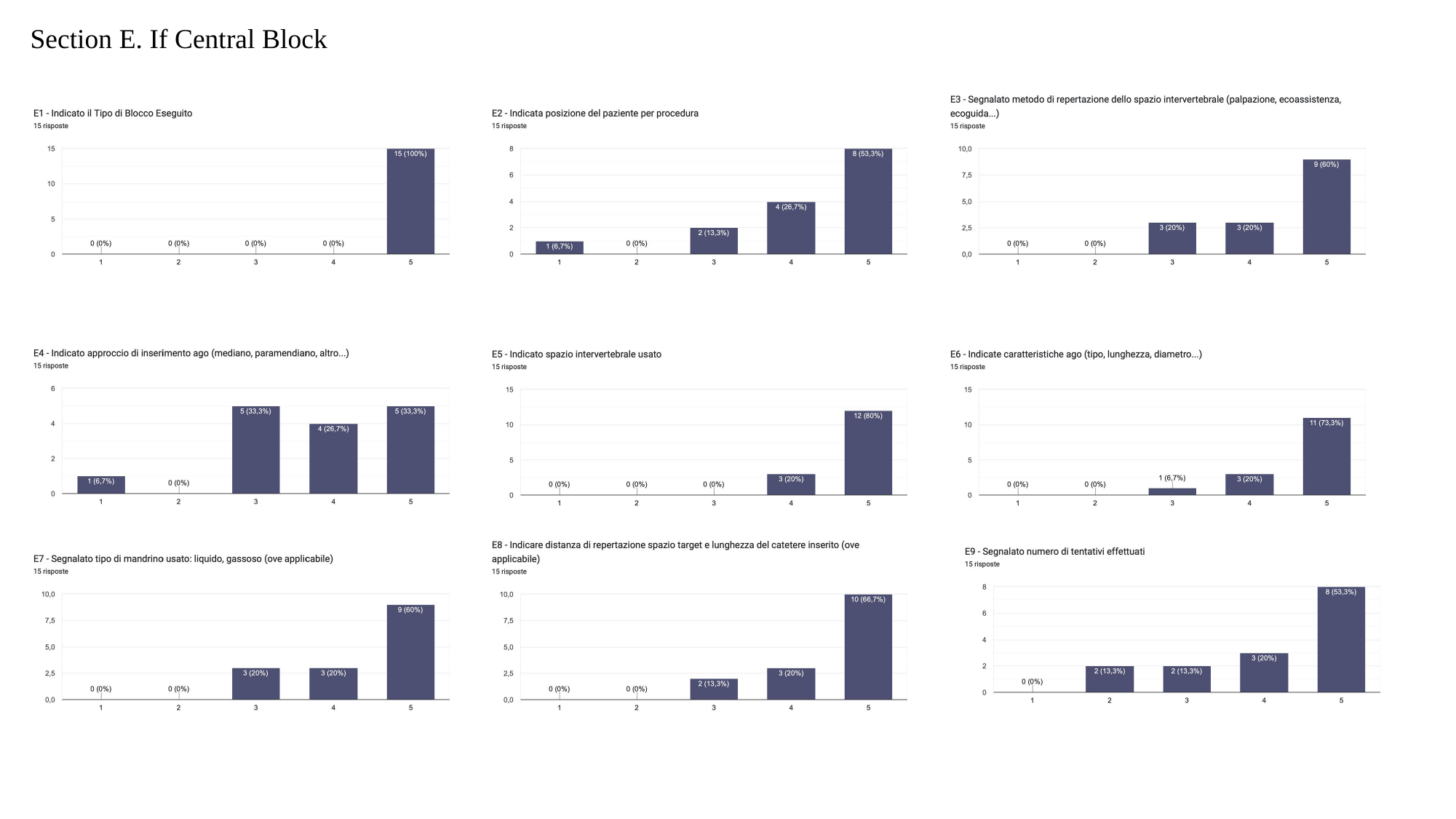

Section E. If Central Block

## Slide 5
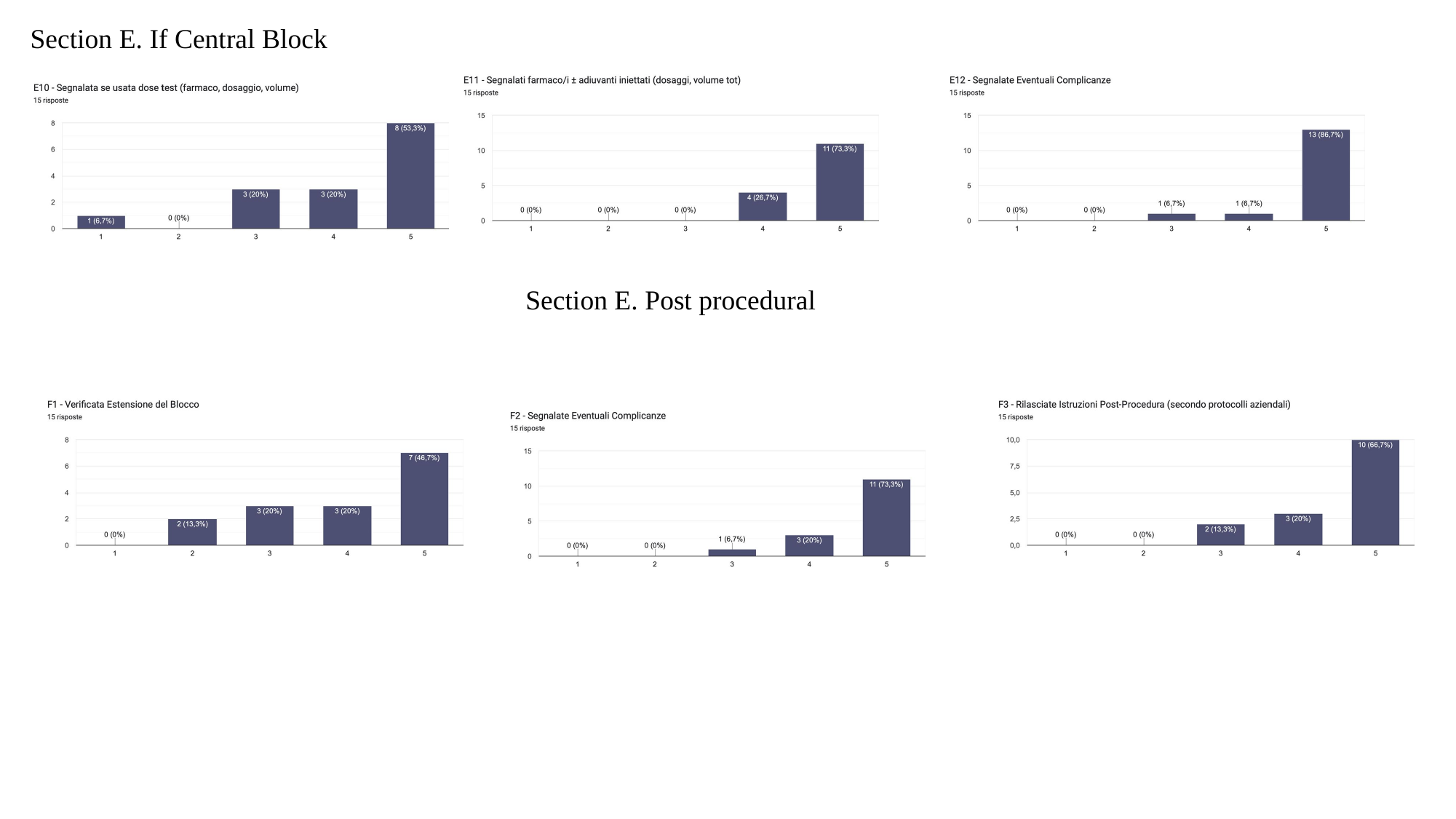

Section E. If Central Block
Section E. Post procedural
